# The complete chloroplast genome of *Tulipa gesneriana* (Liliaceae) and its phylogenetic analysis

**DOI:** 10.1080/23802359.2022.2093676

**Published:** 2022-07-08

**Authors:** Lingli Yuan, Xiaozhi Yan, Xian Chen, Xingfu Zhu

**Affiliations:** School of Life Sciences, Guizhou Normal University, Guiyang, China

**Keywords:** Chloroplast, *Tulipa gesneriana*, phylogenetic analysis

## Abstract

The complete chloroplast genome sequence of *Tulipa gesneriana* L. was determined to investigate its phylogenetic position. This plastome is 151,958 base pairs (bp) in length, and comprises two inverted repeat (IRa and IRb) regions of 26,352 bp, a small single-copy region of 17,123 bp and a large single-copy region of 82,131 bp. The GC contents of the cp genome were 36.6%. In total, we annotated 126 genes including 81 protein-coding genes, 37 tRNA genes, and eight rRNA genes. Phylogenetic analysis based on nine chloroplast genomes indicates that *T. gesneriana* is closely related to *T. iliensis* and *T. thianschanica*.

Tulips (*Tulipa*) are among the world's most well-known, beloved, and economically important ornamental plants due to their large, showy, and various colors flowers (Eijk et al. 1991). Most of the cultivars of tulip are derived from *Tulipa gesneriana* L. 1753 and it has become naturalized in parts of central and southern Europe and scattered locations in North America. Several chloroplast genomes of Tulips have been published, but the cp genome of *T. gesneriana* is lacking (Ju et al. 2020; Li et al. 2021). Due to its ecological and economic importance, the genetic and genomic information is urgently needed to provide fundamental genetic reference for its molecular breeding and biological research. Here, we made the first report of a complete plastome for *T. gesneriana* (GenBank accession number: ON041137).

The total genomic DNA was extracted from dry leaves sampled from Kunming (Yunnan, China, E102.7680, N25.1381) and Voucher herbarium specimens were deposited at the Herbarium of Guizhou Normal University (Xingfu Zhu, zhuxingfu@outlook.com) under the voucher number zhu20200308. Total genomic DNA was extracted with the Qiagen DNeasy Plant Mini Kit (Qiagen, Carlsbad, CA). After cluster generation, the genomic paired-end (PE150) sequencing was performed on an Illumina Hiseq 2000 instrument (Illumina, Inc., San Diego, CA). The genome was assembled using the program GetOrganelle v1.7.5 using the filtered reads (Jin et al. 2020). Annotation was performed using PGA (Qu et al. 2019) and Cpgavas2 (Shi et al. 2019), and then manually corrected.

A typical quadripartite structure as most angiosperms was displayed by the plastome of *T. gesneriana*, containing two inverted repeat regions of 26,352 base pairs (bp), a large single-copy region of 82,131 bp, and a small single-copy region of 17,123 bp. The GC content of the whole complete chloroplast genome was 36.6%. A total of 126 gene functional genes were annotated, including 81 protein-coding genes, eight ribosomal RNA genes, and 37 tRNA genes. In these genes, eight protein-coding genes (atpF, clpP, ndhA, ndhB, rpl2, rps12, rpoC1, and rpoC2) contained one intron and two genes (ycf3 and ycf1) contained two introns. The protein-coding genes, tRNA genes, and rRNA genes account for 64.29, 29.36, and 6.35% of all annotated genes, respectively.

To further investigate the phylogenetic position of *T. gesneriana*, a maximum-likelihood tree was constructed based on eight published complete plastomes of Liliaceae and the new plastome. We constructed the tree using RAxML (Stamatakis 2014) after the sequences were aligned using MAFFT v7.307 (Katoh and Standley 2013). Our results showed that *T. gesneriana* was close to the *T. iliensis* and *T. thianschanica* (Figure 1). This published chloroplast genome will provide useful information for phylogenetic and evolutionary studies in *Tulipa.*

**Figure 1.**
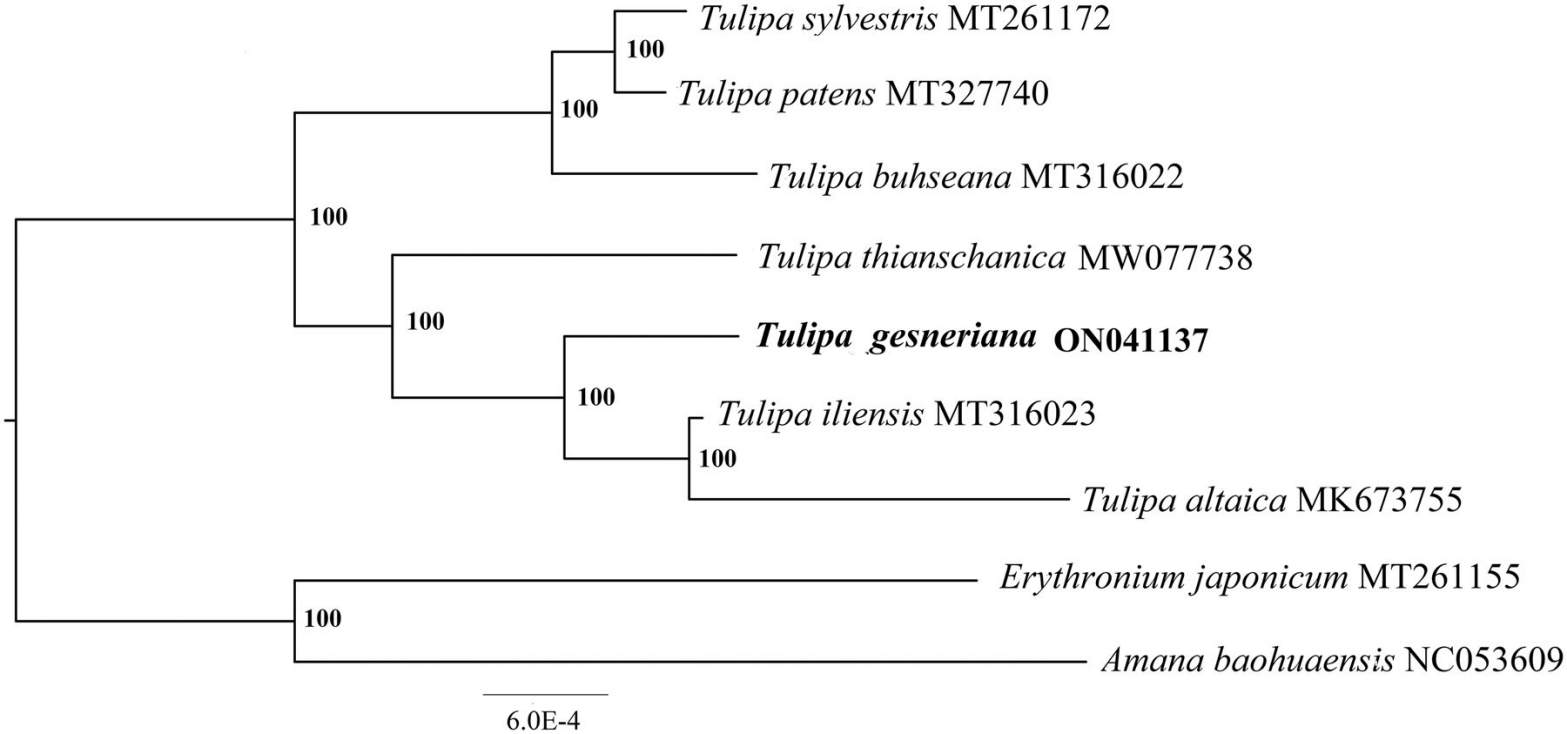
ML phylogenetic tree of nine species within Liliaceae based on eight chloroplast genome sequences in GenBank, plus the chloroplast sequence of *Tulipa gesneriana*. The tree is rooted with the *Amana baohuaensis* and *Erythronium japonicum*. Bootstraps (100,000 replicates) are shown at the nodes.

## Data Availability

The data that support the findings in this study are openly available. The complete chloroplast genome sequence of *T. gesneriana* has been deposited in GenBank with accession number ON041137 (https://www.ncbi.nlm.nih.gov/nuccore/ON041137.1/). The associated BioProject, BioSample, and SRA accession numbers are PRJNA821271, SAMN27064805, and SRX14680869, respectively.

## References

[CIT0001] Eijk J, Raamsdonk L, Eikelboom W, Bino RJ. 1991. Interspecific crosses between *Tulipa gesneriana* cultivars and wild *Tulipa* species: a survey. Sex Plant Reprod. 4(1):1–5.

[CIT0002] Jin J-J, Yu W-B, Yang J-B, Song Y, dePamphilis CW, Yi T-S, Li D-Z. 2020. GetOrganelle: a fast and versatile toolkit for accurate de novo assembly of organelle genomes. Genome Biol. 21(1):241. 32912315 10.1186/s13059-020-02154-5 PMC7488116

[CIT0003] Ju X, Shi G, Hou Z, Wu C, Liu G, Cao C, Tang N. 2020. Characterization of the complete chloroplast genome of *Tulipa iliensis* (Liliaceae). Mitochondrial DNA Part B. 5(3):2362–2363. 33457791 10.1080/23802359.2020.1773333 PMC7783151

[CIT0004] Katoh K, Standley DM. 2013. MAFFT multiple sequence alignment software version 7: improvements in performance and usability. Mol Biol Evol. 30(4):772–780. 23329690 10.1093/molbev/mst010 PMC3603318

[CIT0005] Li J, Price M, Su D-M, Zhang Z, Yu Y, Xie D-F, Zhou S-D, He X-J, Gao X-F. 2021. Phylogeny and comparative analysis for the plastid genomes of five *Tulipa* (Liliaceae). Biomed Res Int. 2021:6648429. 34239930 10.1155/2021/6648429 PMC8235973

[CIT0006] Qu X-J, Moore MJ, Li D-Z, Yi T-S. 2019. PGA: a software package for rapid, accurate, and flexible batch annotation of plastomes. Plant Methods. 15(1):1–12. 31139240 10.1186/s13007-019-0435-7 PMC6528300

[CIT0007] Shi L, Chen H, Jiang M, Wang L, Wu X, Huang L, Liu C. 2019. CPGAVAS2, an integrated plastome sequence annotator and analyzer. Nucleic Acids Res. 47(W1):W65–W73. 31066451 10.1093/nar/gkz345 PMC6602467

[CIT0008] Stamatakis A. 2014. RAxML version 8: a tool for phylogenetic analysis and post-analysis of large phylogenies. Bioinformatics. 30(9):1312–1313. 24451623 10.1093/bioinformatics/btu033 PMC3998144

